# Novel peptide GX1 inhibits angiogenesis by specifically binding to transglutaminase-2 in the tumorous endothelial cells of gastric cancer

**DOI:** 10.1038/s41419-018-0594-x

**Published:** 2018-05-21

**Authors:** Zhijie Lei, Na Chai, Miaomiao Tian, Ying Zhang, Guodong Wang, Jian Liu, Zuhong Tian, Xiaofang Yi, Di Chen, Xiaowei Li, Pengfei Yu, Hao Hu, Bing Xu, Chao Jian, Zhenyuan Bian, Hao Guo, Jinpeng Wang, Shiming Peng, Yongzhan Nie, Niu Huang, Sijun Hu, Kaichun Wu

**Affiliations:** 1 State key Laboratory of Cancer Biology, National Clinical Research Center for Digestive Diseases and Xijing Hospital of Digestive Diseases, Fourth Military Medical University, Xi’an, 710032 Shaanxi Province People’s Republic of China; 2 Department of Radiology, Xjing Hospital of Fourth Military Medical University, Xi’an, 710032 Shaanxi Province People’s Republic of China; 3 Department of Hepatobiliary Surgery, Xjing Hospital of Fourth Military Medical University, Xi’an, 710032 Shaanxi Province People’s Republic of China; 4 Department of Neurosurgery, Tangdu Hospital of Fourth Military Medical University, Xi’an, 710038 Shaanxi Province People’s Republic of China; 5 Department of Orthopedics, Xjing Hospital of Fourth Military Medical University, Xi’an, 710032 Shaanxi Province People’s Republic of China; 6 National Institute of Biological Sciences, Beijing, 102206 People’s Republic of China

## Abstract

The clinical application of GX1, an optimal gastric cancer (GC) targeting peptide, is greatly limited because its receptor in the GC vasculature is unknown. In this study, we screened the candidate receptor of GX1, transglutaminase-2(TGM2), by co-immunoprecipitation (co-IP) combined with mass spectrometry. We found that TGM2 was up-regulated in GC vascular endothelial cells and that GX1 receptor expression was suppressed correspondingly after TGM2 downregulation. A highly consistent co-localization of GX1 receptor and TGM2 was detected at both the cellular and tissue levels. High TGM2 expression was evident in GC tissues from patients with poor prognosis. After TGM2 downregulation, the GX1-mediated inhibition of proliferation and migration and the induction of the apoptosis of GC vascular endothelial cells were weakened or even reversed. Finally, we observed that GX1 could inhibit the GTP-binding activity of TGM2 by reducing its intracellular distribution and downregulating its downstream molecular targets (nuclear factor-kappa B, NF-κB; hypoxia-inducible factor 1-α, HIF1α) in GC vascular endothelial cells. Our study confirms that peptide GX1 can inhibit angiogenesis by directly binding to TGM2, subsequently reducing the GTP-binding activity of TGM2 and thereby suppressing its downstream pathway(NF-κB/HIF1α). Our conclusions suggest that GX1/TGM2 may provide a new target for the diagnosis and treatment of GC.

## Introduction

Since Folkman first determined that the growth of solid tumors comprising diameters above several millimeters depended on angiogenesis^1^, increasing research has addressed tumor vascular-targeted therapy^2,3^. Peptides have few interactions with the immune system, good tumor and tissue penetration abilities and the potential for large-scale production and reproducibility, and thus, they have become the optimal therapeutic agent for targeting tumor blood vessels^4,5^. The phage display library has enabled the screening and identification of numerous peptides that specifically home to the tumor vasculature^6^.

A cyclic 7-mer peptide, namely GX1 (CGNSNPKSC), that targeted the human gastric cancer (GC) vasculature was identified by screening a Ph.D.-C7C phage display library in vivo^7^. Immunohistochemistry analysis of murine and human tissues showed that GX1 could specifically bind to the endothelial cells of human GC^8^. GX1 labeled with Cy5.5, ^64^Cu and ^99^Tc^m^ was also robustly targeted to GC both in vitro and in vivo^8–10^. Ben Chen demonstrated that GX1 could inhibit vascular endothelial cell proliferation by inducing apoptosis in vitro and neovascularization in vivo^11^. Furthermore, after recombination, GX1-rmhTNF-α became more selective and exhibited elevated anti-tumor activity with decreased systemic toxicity^11^. Overall, the abovementioned studies confirm that GX1 has the potential to serve as a homing peptide in vascular targeted therapy for GC.

However, because the receptor of GX1 in GC is unknown, the extensive clinical therapeutic applications of GX1 are limited. Hence, screening for downstream targets of GX1 and exploring the mechanism through which GX1 inhibits angiogenesis are of utmost importance.

In this study, after repeated co-immunoprecipitation (co-IP) and mass spectrometric analyses, we eventually identified a candidate protein, transglutaminase-2 (TGM2), as the receptor through which GX1 targets the GC vasculature. TGM2, a 76-kDa, member of the transglutaminase family that is primarily localized to the cytosol and plasma membrane, is an inducible transamidating acyltransferase that catalyzes Ca^2+^-dependent protein modifications and plays fundamental roles in cellular differentiation and apoptosis^12–14^. Kumar’s study shows that intracellular TGM2 constitutively activates NF-κB and vice versa and that NF-κB activates HIF1 and vice versa. Coincidentally, the VEGF gene is a downstream target of HIF1α. As a result, one can conceive that TGM2 promotes angiogenesis by activating HIF1α to subsequently induce VEGF expression^15,16^. Recent studies have revealed that elevated TGM2 expression is a factor in tumor survival^17^. Regarding the role of TGM2 in GC, Xiaofeng Wang observed that TGM2 was highly expressed in GC tissues; that promoted GC cell proliferation, migration, and invasion; and that TGM2 promoted tumorigenesis and peritoneal metastasis in vivo^18^.

## Results

### Co-HUVECs (co-cultured human umbilical vein endothelial cells and human gastric adenocarcinoma cells SGC-7901) showed high expression of GX1 receptor and GX1 can inhibit the migration ability of co-HUVECs

**Fig. 1:**
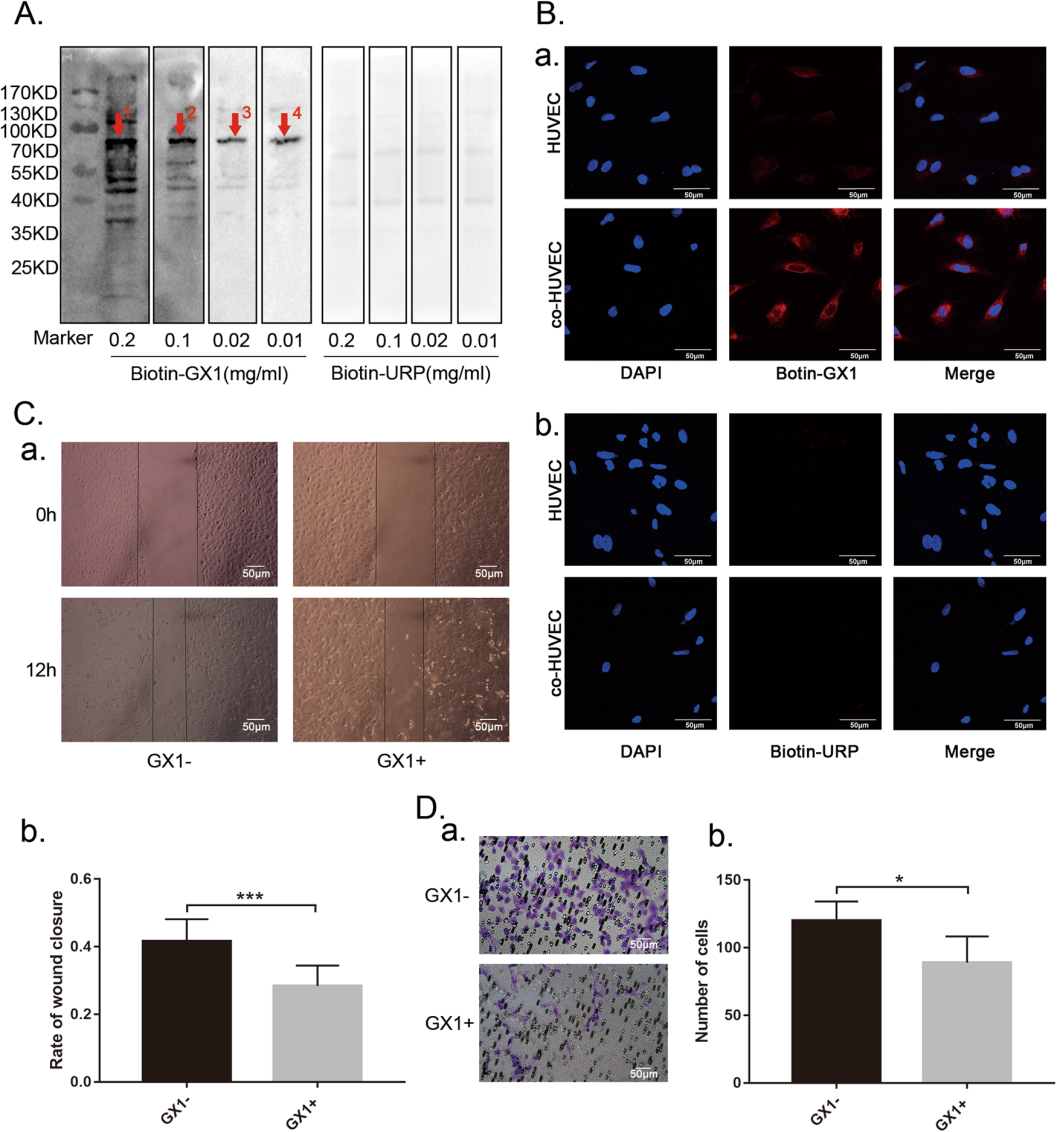
Detection of GX1-receptor expression in co-HUVECs and the effect of GX1 on co-HUVECs migration. **a** After co-HUVEC lysates were incubated with different concentrations of biotin-GX1 and biotin-URP (0.2, 0.1, 0.02, and 0.01 mg/ml), one specific binding band at approximately 70 kDa was detected with the biotin-labeled peptide GX1 by Western blotting (indicated by red arrows 1, 2, 3, and 4). **b** Immunofluorescence staining of the GX1 receptor (red) in HUVECs and co-HUVECs. **c** Wound healing assay of the migration of co-HUVECs pretreated with or without GX1. Pictures were obtained at a magnification of 200× and 12 h after scratching. ***P < 0.001, *n* = 4. **d** Transwell assay examining the effect of GX1 (0.1 mg/ml, 24 h) on co-HUVECs migration. Pictures were captured at a magnification of 200×. The number of migratory cells per field was calculated with ImageJ software. *P < 0.05, *n* = 4

To detect the expression of GX1-receptor, we incubated co-HUVECs lysates with different concentrations of biotin-GX1 (biotinylated peptide GX1) solution (0.2, 0.1, 0.02, and 0.01 mg/ml) and with biotin-URP (biotinylated unrelated peptide) as a negative control. The Western blotting results indicated that one specific band at approximately 70 kDa, could be consistently identified in co-HUVECs lysates (Fig. 1a, indicated by red arrows 1–4) and that the band became clearer with decreasing concentrations of biotin-GX1. In contrast, no band was detected at the corresponding size in the biotin-URP control lysates (Fig. 1a). The cell immunofluorescence staining results showed that GX1-receptor was highly expressed in co-HUVECs compared with HUVECs and that GX1-receptor was primarily localized to the cell membrane and cytosol (a, Fig. 1b). However, the negative control URP-receptor was expressed weakly in both HUVECs and co-HUVECs (b, Fig. 1b). The wound healing (Fig. 1c, ***P < 0.001) and Transwell (Fig. 1d, *P < 0.05) assays indicated that GX1 (0.1 mg/ml, 24 h) could suppress the migratory ability of co-HUVECs, and the differences were statistically significant.

### GX1 receptor is identified as TGM2 by co-IP and mass spectroscopic analysis

**Fig. 2:**
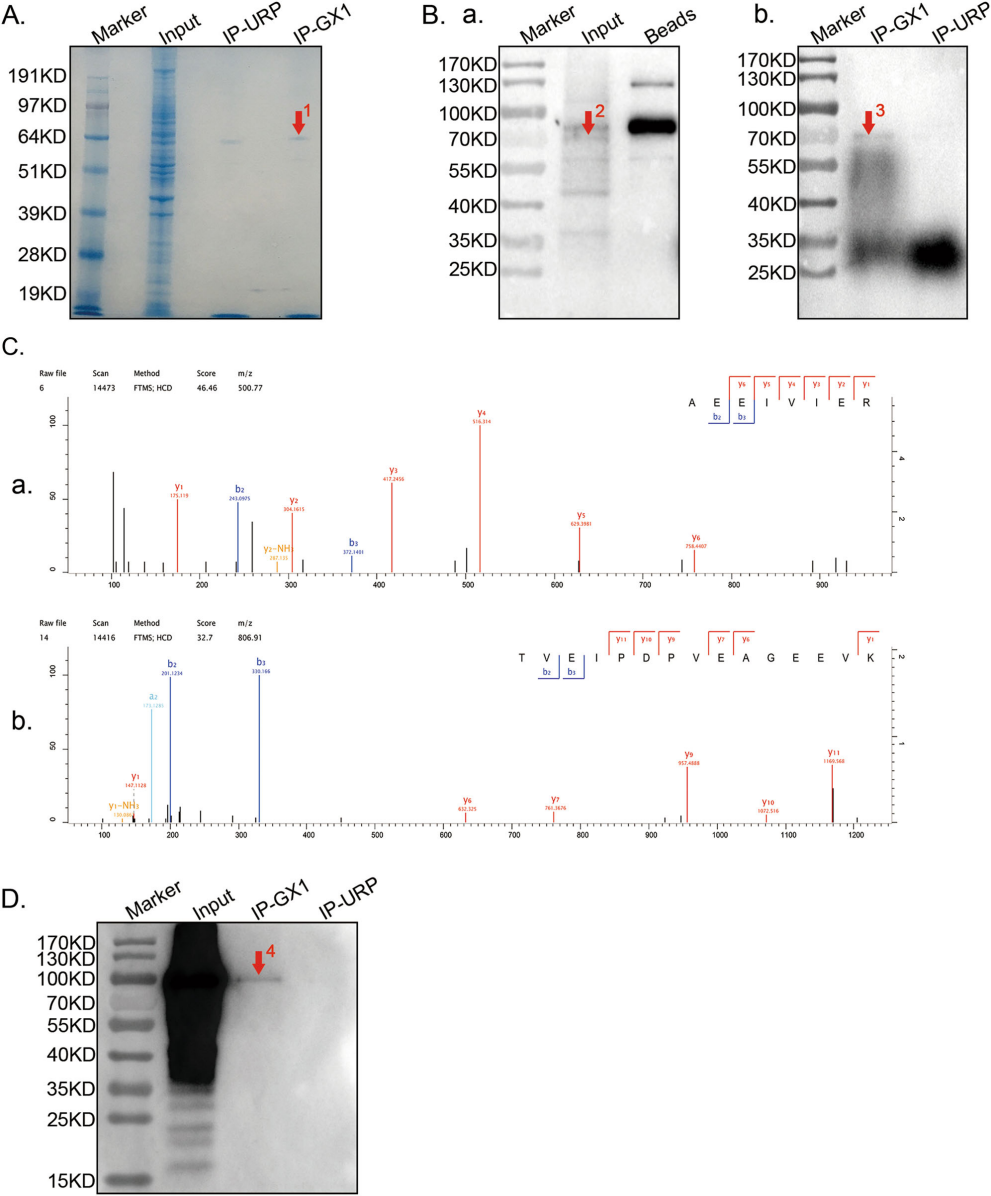
Screening the receptor of GX1 in co-HUVECs. **a**, **b** Proteins that bound to GX1 were enriched from co-HUVEC lysates by co-immunoprecipitation, separated by SDS-PAGE, subjected to Coomassie blue staining (**a**) and incubated with biotin-GX1(**b**). One immune-reactive band was detected at approximately 70 kDa in both the input (indicated by red arrow 2) and IP-GX1 lane (protein binding to GX1, indicated by red arrows 1 and 3). The IP-URP (protein binding to URP) and Beads (protein binding to magnetic beads) lanes were used as negative controls. **c** (panels a and b) TGM2 was identified by Q-Exactive mass spectrometry. The MS/MS spectra of TGM2 that were obtained by Q-Exactive, coupled with nanoflow capillary high-performance liquid chromatography after trypsin digestion of spot 1 (**a**) is shown. **d** Protein binding to GX1 during co-immunoprecipitation was separated by SDS-PAGE and subsequently incubated with TGM2-antibody. Results of Western blotting showed that a specific band was detected in IP-GX1 (approximately 100 kDa, indicated by red arrow 4) lane and that no band was detected at the same position in IP-URP lane

To identify the GX1 receptor, co-IP was conducted. Protein binding to GX1 (IP-GX1) was determined by pulling down bead-streptavidin-biotin-GX1 complexes from co-HUVECs lysates. Protein binding to the bead-streptavidin-biotin-URP complexes (IP-URP) and protein binding to the beads (Beads) were set as negative controls. Western blotting and Coomassie blue staining were performed to examine the extent of enrichment of GX1-receptor. Similar to GX1-receptor expression in the co-HUVECs lysate (Fig. 1a), we observed a clear band at ~70 kDa in both the Coomassie blue staining results (Fig. 2a, indicated by arrow 1) and Western blotting results (Fig. 2b, indicated by arrows 2 and 3) in the input and IP-GX1 lanes. Nevertheless, no band was evident at the corresponding size in the IP-URP (b, Fig. 2b) or Beads (a, Fig. 2b) groups. The corresponding protein band was excised from the Coomassie blue-stained gel and subjected to in-gel tryptic digestion and mass spectroscopic analysis. Next, 55 candidate proteins that might be receptors for GX1 in co-HUVECs were detected in the corresponding protein band (Supplementary Table 1). Based on the GX1-receptor molecular weight and specifically binding to GX1 but not URP (Supplementary Table 2), we ultimately chose TGM2 (Fig. 2c) as the top candidate receptor of GX1; however, further confirmation of this candidate was needed. Next, proteins binding to GX1 from co-immunoprecipitation were separated by SDS-PAGE and subsequently incubated with TGM2-antibody. Results of western blotting showed a unique band in the IP-GX1 lane and no band at the corresponding size in IP-URP lane, indicating a specific interaction between the GX1-receptor and TGM2-antibody (Fig. 2d).

### TGM2 expression is detected and TGM2 is co-localized with GX1 receptor in GC vascular endothelial cells

**Fig. 3:**
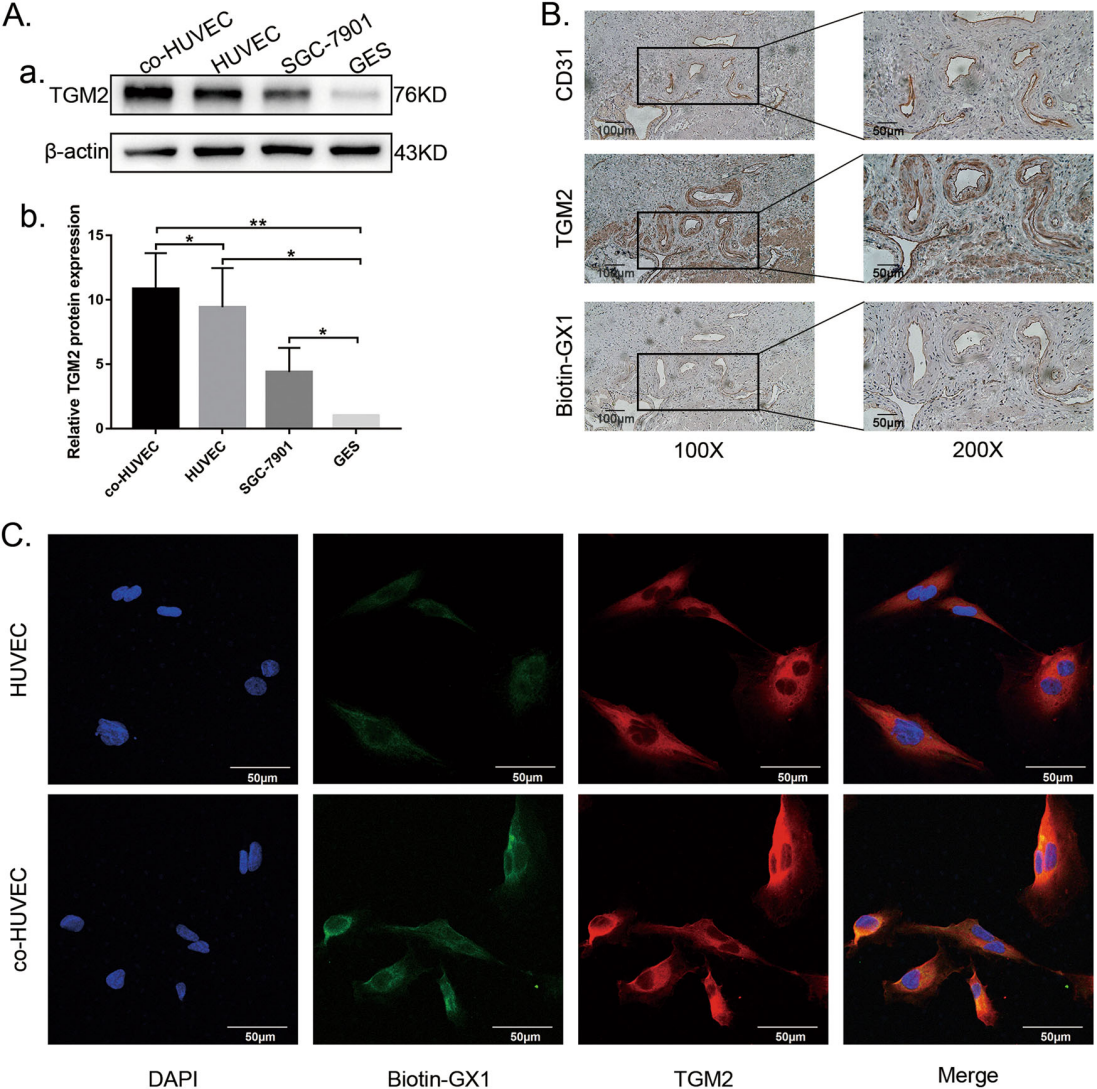
Expression of TGM2 and co-localization of GX1-receptor and TGM2 in cells and tissue of gastric cancer. **a** (panels a and b) Total protein lysates from co-HUVECs, HUVECs, SGC7901 cells and GES cells were incubated with TGM2-antibody and examined by Western blotting. *P < 0.05; **P < 0.01, *n* = 3. **b** Three serial sections of gastric cancer tissues were analyzed concurrently by immunohistochemistry staining for GX1-receptor, TGM2 or CD31. **c** Dual immunofluorescence staining of GX1-receptor (green) and TGM2 (red) in HUVECs and co-HUVECs. The Merged pictures show the overlay of GX1-receptor (green) and TGM2 (red) and the nuclei stained with 40, 6-diamidino-2-phenylindole (DAPI, blue)

To evaluate TGM2 expression and its co-localization with GX1-receptor, western blotting, immunofluorescence and immunohistochemistry staining were conducted. Western blotting analysis of TGM2 expression in co-HUVECs, HUVECs, SGC-7901 cells and gastric mucosal epithelial (GES) cells showed that TGM2 expression was higher in co-HUVECs than in HUVECs or GES (Fig. 3a, **P < 0.01). TGM2 expression was lower in GES cells than in SGC-7901 cells, and TGM2 expression in GES cells was the lowest of all four cell lines (Fig. 3a, *P < 0.05; **P < 0.01). Merged pictures of dual immunofluorescence staining in HUVECs and co-HUVECs show the consistent subcellular localization of GX1-receptor and TGM2 and that the expression of TGM2 is higher in co-HUVECs than in HUVECs (Fig. 3c). Three serial sections of a GC tissue sample were subjected to concurrent immunohistochemistry staining for GX1-receptor, TGM2 and CD31. The results revealed elevated TGM2 levels in GC vessels and that CD31, TGM2 and GX1-receptor were all localized to GC vessels (Fig. 3b). These results demonstrate the consistent co-localization of GX1-receptor and TGM2 at both the cellular and tissue levels.

### TGM2 is significantly up-regulated in gastric cancer, and high TGM2 expression indicates poor prognosis of GC

**Fig. 4:**
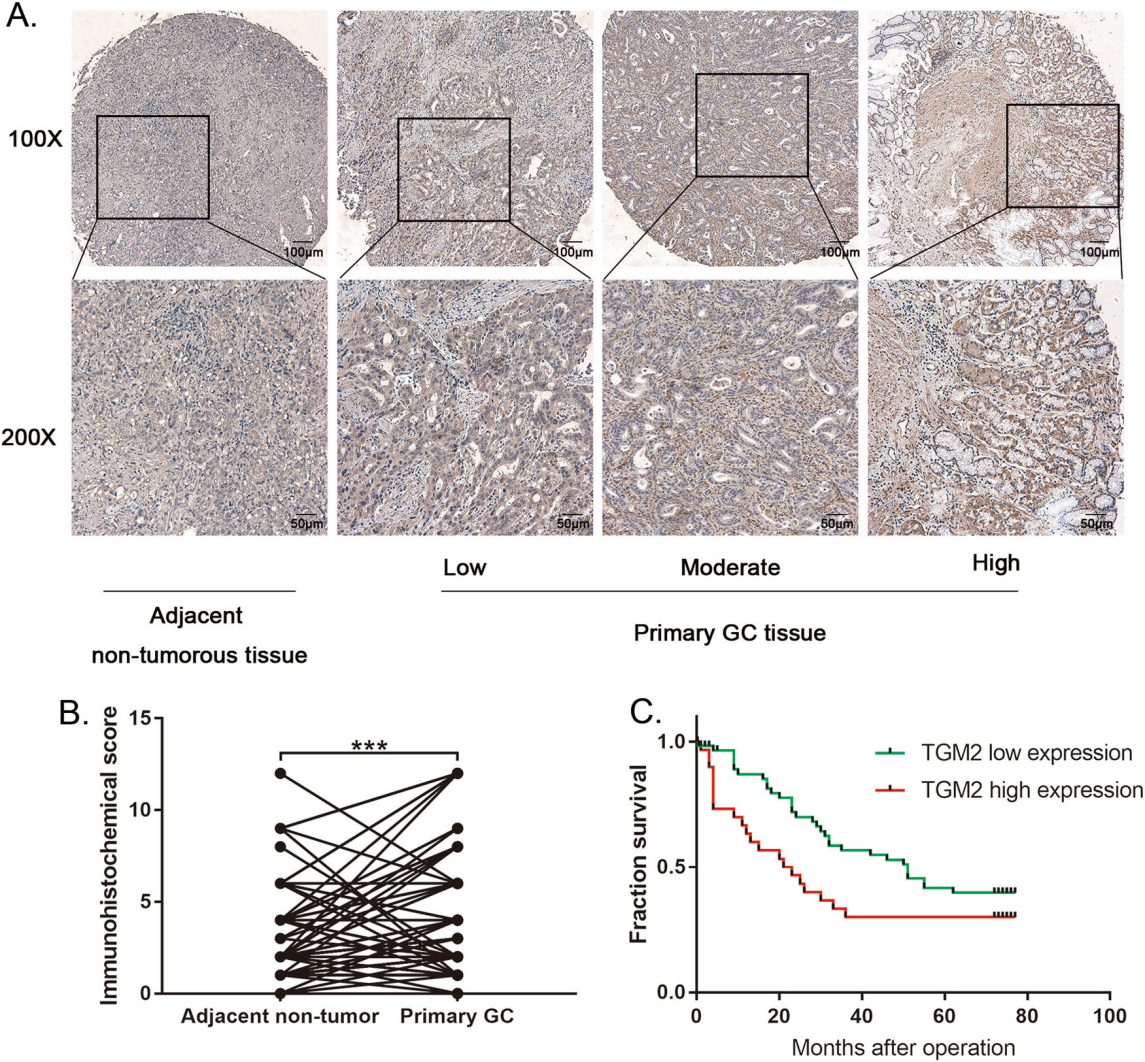
TGM2 is significantly up-regulated in gastric cancer, and high TGM2 expression indicates poor prognosis. **a** Representative TGM2 expression in adjacent non-tumorous tissues and primary GC tissues was detected with immunohistochemistry methods. **b** Comparison of TGM2 expression in primary GC tissues and adjacent non-tumor tissues. **c** Kaplan–Meier analysis of the correlation between TGM2 expression and the fraction survival of human GC patients

To explore the function of TGM2 in determining the clinical outcomes of GC patients, we examined its expression in 90 GC patients with a tissue microarray. Our immunohistochemistry results showed that TGM2 protein expression was significantly higher in GC tissues than in non-tumorous tissues (Figs. 4a, b). The high expression of TGM2 was significantly correlated with the maximum tumor size and a higher tumor-nodule-metastasis stage (Table 1). The Kaplan-Meier analysis indicated that the patients with high TGM2 expression had a shorter survival rate than the patients with low TGM2 expression (Fig. 4c).

**Table 1:** Correlation between TGM2 expression and clinicopathological parameters in GC tissue

Clinicopathological parameters		*n*	TGM2 expression		*P*-value
			Low(*n* = 48)	High(*n* = 42)	
Age(years)	≤60	28	11	17	0.073
	>60	62	37	25	
Gender	Male	68	34	34	0.265
	Female	22	14	8	
Tumor size	≤50	58	38	20	0.002*
	>50	32	10	22	
Degree of differentiation	I/II	29	18	11	0.252
	III	61	30	31	
Vessels invasion	No invasion	74	41	33	0.397
	Invasion	16	7	9	
TNM stage	I/II	41	27	14	0.029*
	III/IV	49	21	28	

*P < 0.05

### TGM2 is functionally essential for the proliferation and migration of co-HUVECs

**Fig. 5:**
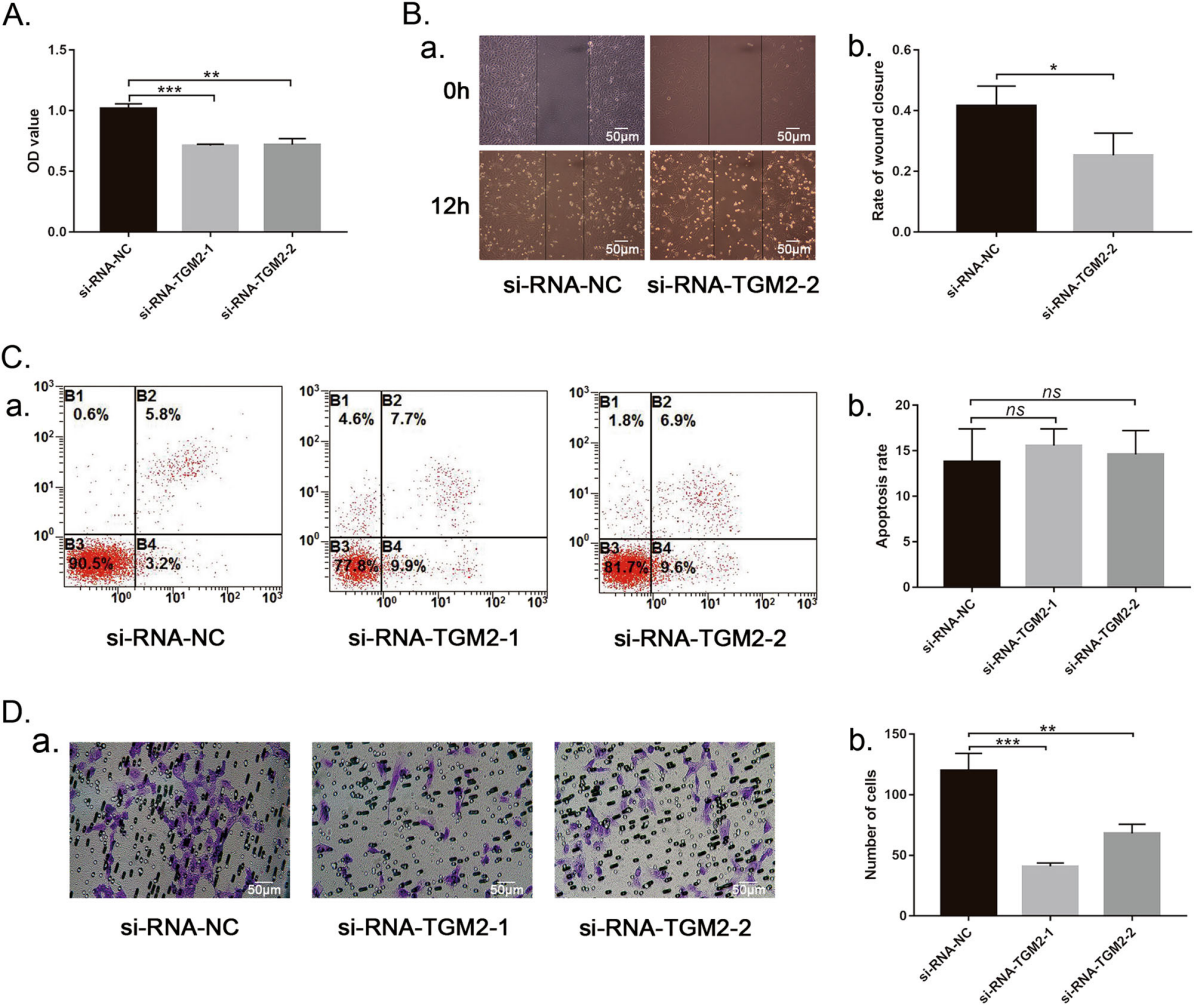
Effect of TGM2 on the proliferation, apoptosis and migration of co-HUVECs. **a** Cell Counting Kit-8 (CCK-8) test to analyze co-HUVEC proliferation with or without TGM2 downregulation. **P < 0.01; ***P < 0.001, *n* = 6. Wound healing (**b**) and Transwell (**d**) assays of co-HUVEC migration after TGM2 downregulation with siRNAs. *P < 0.05; **P < 0.01; ***P < 0.001, *n* = 4. **c** Flow cytometry analysis of co-HUVEC apoptosis after TGM2 knockdown

After the downregulation of TGM2 with siRNAs, the proliferation (Fig. 5a, **P < 0.01; ***P < 0.001) and migration (Fig. 5b, *P < 0.05; and Fig. 5d, **P < 0.01; ***P < 0.001) of the co-HUVECs were significantly suppressed compared to those of controls. The apoptosis rate of the TGM2-downregulated co-HUVECs increased to a certain extent, but no significant difference was evident compared with the control (Fig. 5c, *P* > 0.05). These results suggested close relationships between TGM2 and the proliferation and migration of co-HUVECs.

### TGM2 knockdown reduces GX1-recptor expression as well as GX1 effect on the apoptosis and migration of co-HUVECs

**Fig. 6:**
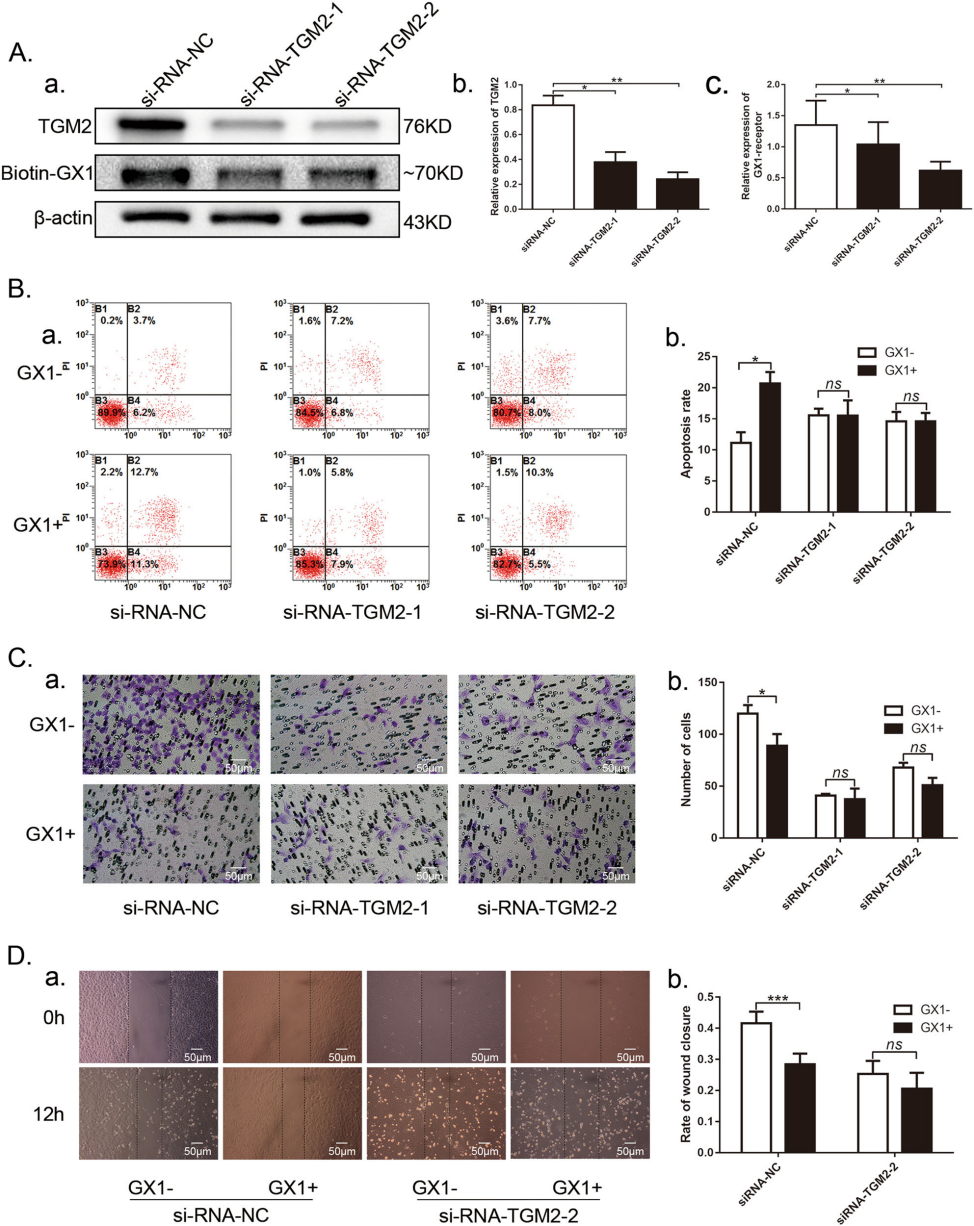
Effect of GX1 on co-HUVEC apoptosis and migration after TGM2 downregulation with siRNAs. **a** (panels a, b, c) Western blotting analysis of GX1-receptor and TGM2-antibody in TGM2 knockdown co-HUVECs. *P < 0.05; **P < 0.01; *n* = 3. **b** Flow cytometry analysis of TGM2 knockdown apoptotic co-HUVECs treated with or without GX1 (0.1 mg/ml, 48 h). Transwell (**c**) and wound healing (**d**) assays to measure the alterations of GX1-mediated inhibition of co-HUVECs migration after TGM2 downregulation. *P < 0.05; ***P < 0.001; *n* = 4

After TGM2 knockdown (b, Fig. 6a, *P < 0.05; **P < 0.01), the expression of GX1-receptor in co-HUVECs was correspondingly suppressed (c, Fig. 6a, *P < 0.05; **P < 0.01). Next, co-HUVECs with or without TGM2 downregulation were pre-incubated with or without GX1 (0.1 mg/ml, 48 h). Under control conditions, GX1 induced the apoptosis of co-HUVECs, but when TGM2 was downregulated with siRNAs (Fig. 6b, *P < 0.05), no significant difference in the apoptosis rate was evident between the co-HUVECs with GX1 incubation and those without GX1 incubation (Fig. 6b, *P* > 0.05). Similarly, when TGM2 expression was suppressed with siRNAs, the inhibitory effect of GX1 (0.1 mg/ml, 24 h) on the migratory ability of co-HUVECs was weakened, and cellular migration even recovered to normal levels (Fig. 6c, *P* > 0.05; Fig. 6d, *P* > 0.05). The reduction in GX1-mediated apoptosis and the reversal of GX1-mediated inhibition of co-HUVEC migration after TGM2 downregulation demonstrated that GX1-mediated effect on co-HUVECs was likely dependent on TGM2, emphasizing a critical interaction between TGM2 and GX1.

### TGM2 intracellular distribution and GTP binding activity is inhibited by GX1 in accompany with the decreasing expression of the downstream molecules (NF-κB and HIF1α) in co-HUVECs

**Fig. 7:**
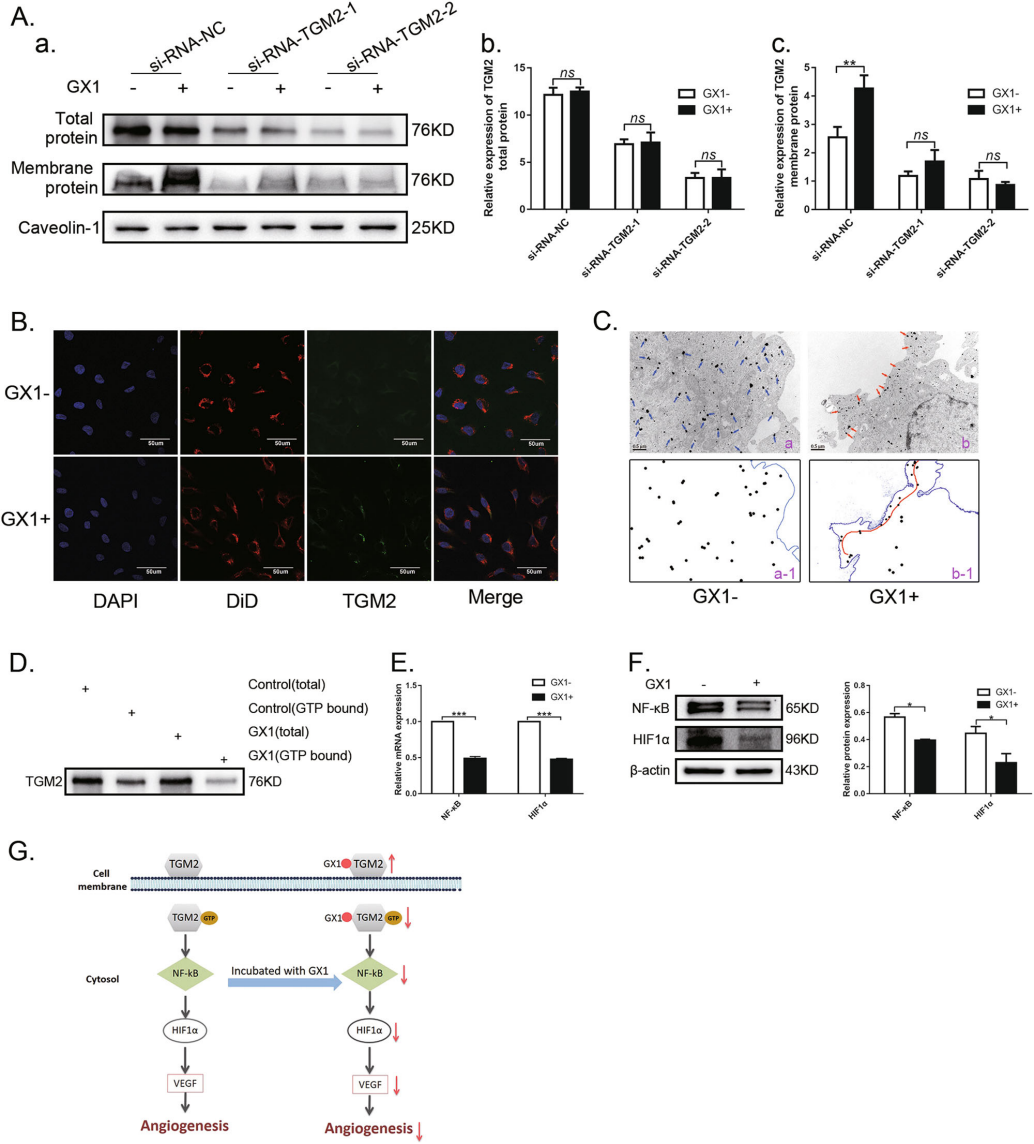
Evaluation of GX1 effect on the TGM2 subcellular distribution, TGM2 G-protein activity and the expression of intracellular-TGM2 downstream molecular targets. **a** Western blotting analysis of TGM2 in total protein samples and membrane protein samples of co-HUVECs treated with or without GX1 (0.1 mg/ml, 24 h) when TGM2 expression was downregulated. **P < 0.01; *n* = 3. **b** The GX1 (0.1 mg/ml, 24 h) effect on the subcellular distribution of TGM2 in co-HUVECs by immunofluorescence. **c** The GX1 (0.1 mg/ml, 24 h) effect on the subcellular distribution of TGM2 in co-HUVECs by immunoelectron microscopy (a-1 represents a schematic of a; b-1 represents a schematic of b). The TGM2 distribution in the control was indicated by blue arrows. The TGM2 distribution in co-HUVECs treated with GX1 was indicated by red arrows. **d** Alteration of the GTP-binding activity of TGM2 in co-HUVECs treated with GX1 (0.1 mg/ml, 24 h). **e** The GX1 (0.1 mg/ml, 24 h) effect on the mRNA expression of NF-κB and HIF1α in co-HUVECs. ***P < 0.001; *n* = 3. **f** The GX1 (0.1 mg/ml, 24 h) effect on the expression of NF-κB and HIF1α at the protein level in co-HUVECs. *P < 0.05; *n* = 3. **g** A schematic illustration of the mechanism through which GX1 inhibits angiogenesis in gastric cancer

To explore the specific mechanism through which GX1 interacts with TGM2, we first determined the effect of GX1 on the total protein expression levels of TGM2 in co-HUVECs. The results showed no significant differences (b, Fig. 7a, *P* > 0.05). Next, we isolated the membrane proteins of co-HUVECs. TGM2 protein expression in the membranes of co-HUVECs with GX1 incubation (0.1 mg/ml, 24 h) was significantly increased compared with that in the membranes of co-HUVECs without GX1 incubation (c, Fig. 7a, **P < 0.01). However, when TGM2 was downregulated with siRNAs, significant differences were not observed between the co-HUVECs with GX1 treatment (0.1 mg/ml, 24 h) and those without GX1 treatment (c, Fig. 7a, *P* > 0.05). To identify which subcellular membrane compartment TGM2 was enriched upon GX1 treatment, the cell membrane of co-HUVECs was labeled with DiD (1,1’-Dioctadecyl-3,3,3',3’-tetramethylindodicarbocyanine perchlorate, a cell membrane fluorescent probe), and then the result of immunofluorescence showed that TGM2 and DiD could be well merged in co-HUVECs treated with GX1, which visually indicated that TGM2 was enriched in the cell membrane of co-HUVECs treated with GX1 (Fig. 7b). At the same time, suppression of intracellular TGM2 distribution in co-HUVECs upon GX1 treatment was observed by immunoelectron microscopy (Fig. 7c). For intracellular TGM2 mainly acts as G-protein, we next measured the GTP-binding activity of TGM2 and found that the GTP-binding activity was decreased in co-HUVECs treated with GX1 (Fig. 7d). Simultaneously, the downregulation of the downstream molecular targets of intracellular TGM2 (NF-κB and HIF1α) was observed by qRT-PCR (Fig. 7e, ***P < 0.001) and western blotting (Fig. 7f, *P < 0.05). Further study showed that GX1 could inhibit the expression of NF-κB and HIF1α with dose-dependent effects (Supplementary Figure 2). In conclusion, these results demonstrated that GX1 could inhibit the GTP-binding activity of TGM2 by reducing its intracellular distribution and that subsequently downregulate its downstream molecules (NF-κB and HIF1α) in co-HUVECs (Fig. 7g).

## Discussion

The targeting of tumors by traditional tumor vascular targeting agents, such as bevacizumab and aflibercept^2^, is less than ideal, and thus, these agents are associated with serious side effects, including hypertension and renal and cardiac toxicity in a substantial proportion of patients^19^. Because of their smaller molecular weights, stronger targeting abilities, and better tumor tissue penetration levels, peptides have been widely regarded as optimal choices for tumor targeted therapy, such as peptide RGD and NGR^20–22^, which have been widely used in tumor treatment and diagnostics. Previous results have shown that GX1-receptor is highly expressed in the vascular endothelium of GC and that GX1-receptor expression is negatively correlated with the degree of tumor differentiation^8^. In vitro and in vivo experiments have confirmed that GX1 inhibits angiogenesis, and that GX1-rmhTNF-α enhanced the inhibition of tumor growth with a reduction in systemic side effects. However, because the mechanism of interaction between GX1 and GC vascular endothelial tissues is unclear, further clinical applications of GX1 have been greatly limited.

GX1-specific binding proteins were enriched from co-HUVECs lysate by co-IP. Western blotting and Coomassie brilliant blue staining showed that the enriched proteins appeared as an ~70 kDa band. Next, the enrichment bands were analyzed by mass spectrometry. According to specificity, molecular weight, and repeatability, we ultimately identified TGM2 as the candidate receptor of GX1. Previous studies have shown that TGM2 is closely related to angiogenesis, and intracellular TGM2 in complex with the p65 subunit of NF-κB binds to the HIF1 promoter to induce HIF1 expression even under normoxic conditions. The transcriptional activity of TGM2-induced HIF1α was confirmed with HIF1α-response elements from the VEGF promoter, using luciferase as the reporter system. Because the VEGF gene is a downstream target of HIF1α, one can logically presume that TGM2 promotes angiogenesis by activating HIF1α, subsequently inducing VEGF expression^16^.

Western blotting analysis showed that TGM2 was highly expressed in GC vascular endothelial cells (co-HUVECs) compared to GC cells (SGC-7901) and GES. Furthermore, GX1-receptor expression in GC vascular endothelial cells was correspondingly suppressed upon TGM2 downregulation with siRNAs. Immunofluorescence assays at the cellular level showed that both GX1-recptor and TGM2 were co-localized to the cytoplasm and membrane and could be well merged. Immunohistochemistry of GC tissues showed the association between high TGM2 expression and poor prognosis. Moreover, TGM2 expression was especially significant in GC vascular tissue. The staining of biotin-GX1, CD31 and TGM2 showed a high degree of consistency in different serial sections of the same GC tissue. A previous study showed that GX1 could induce the apoptosis of GC endothelial cells^11^. Therefore, we downregulated TGM2 expression and observed that the GX1 induction of apoptosis was weakened or even reversed. We also observed that GX1 could inhibit the migration of GC endothelial cells. Likewise, this GX1-mediated inhibition of migration was weakened or even reversed after TGM2 downregulation. These results demonstrate that GX1 interacts with GC vascular tissue by specifically recognizing TGM2 in GC vascular endothelial cells.

A previous study suggested that GX1 might up-regulate the expression of caspase3 to induce the apoptosis of GC vascular endothelial cells^11^. However, the mechanism through which GX1 interacts with TGM2 to inhibit angiogenesis remains unknown. Hence, we conducted a preliminary investigation. First, we examined the total protein expression levels of TGM2 in GC vascular endothelial cells with GX1 incubation, and our results showed that GX1 had no significant effect on the total protein expression of TGM2. Next, we extracted the membrane protein from co-HUVECs lysates and Western blotting analysis showed that the expression of TGM2 membrane proteins in co-HUVECs with GX1 incubation was significantly up-regulated compared with that in co-HUVECs without GX1 incubation, which indirectly indicated that the distribution of intracellular TGM2 was altered. Suppression of the intracellular TGM2 distribution was observed by immunofluorescence and immunoelectron microscopy. A previous study showed that intracellular TGM2 mainly functioned as a GTP/GDP binding/signaling protein/GTPase^23,24^. Thus, we measured the GTP-binding activity of TGM2 and found that the GTP-binding activity of TGM2 was decreased in co-HUVECs treated with GX1. Next, the mRNA and protein levels of the downstream molecular targets of intracellular TGM2 (NF-kB and HIF1α) were downregulated in co-HUVECs with GX1 treatment. These results demonstrate that GX1 can inhibit the GTP-binding activity of TGM2 by reducing its intracellular distribution and that subsequently downregulate its downstream molecular targets (NF-κB and HIF1α) in co-HUVECs.

In conclusion, our study confirms that Peptide GX1 could inhibit angiogenesis by directly binding to TGM2, subsequently reducing the GTP-biding activity of TGM2 and thereby suppressing its downstream pathway(NF-κB/HIF1α). GX1/TGM2 may provide a new target for the diagnosis and treatment of GC. However, the specific binding site through which GX1 binds to TGM2 remains unclear. A previous study has demonstrated that inhibitors (NC9, VA4 and VA5) that specifically bind to the TGM2 transamidase site can shift the conformational equilibrium of TGM2 to favour the open conformation and that this conformation change disorganizes the GTP-binding site to reduce GTP binding and GTP-related TGM2 signaling^23^. Whether GX1 plays a similar role to that of the inhibitors (NC9, VA4, and VA5) needs further study.

## Materials and methods

### Cell lines and cell culture

Human umbilical vein endothelial cells (HUVECs) were purchased from ScienCell Research Laboratories (Catalog Number: 8000, USA). Human immortalized gastric mucosal cells (GES) and gastric adenocarcinoma cancer cells (SGC-7901) were preserved in our institute. HUVECs were cultured in endothelial cell matrix (ECM, ScienCell Research Laboratories, Catalog Number: 1001, USA) with 10% fetal bovine serum (FBS, ScienCell Research Laboratories, Catalog Number: 0025, USA), 1% endothelial cell growth supplement (ECGS, ScienCell Research Laboratories, Catalog Number:1052, USA), and 1% penicillin/streptomycin solution (P/S, ScienCell Research Laboratories, Catalog Number: 0503, USA). GES and SGC-7901 were cultured in RPMI-1640 medium (Gibco, Catalog Number: 11875119, USA) with 10% FBS (Gibco, Catalog Number: 10099-141, USA), 1% P/S (HyClone, Catalog Number: SV30010, USA). Model of co-HUVECs (co-culture of HUVECs and SGC-7901) was constructed to simulate GC vasculature in vitro as described^11,25^. Tumor conditioned medium (TCM) was prepared by incubating SGC-7901 cells (1 × 10^6^/ml) in ECM (free of FBS and ECGS) for 24 h. The medium was subsequently removed, centrifuged (2000×*g*, 10 min), filtered through a 0.22-μm filter and diluted five times with ECM supplemented with FBS and EGCS. Tumor endothelial cells were generated by incubating HUVECs in TCM^26^. All cells were incubated at 37 °C with 5% CO_2_ in a humidified incubator.

### Synthesis of peptides

GX1 (CGNSNPKSC), URP (unrelated peptide, cyclo-peptide CNKSPSGNC), biotin-GX1 (biotinylated peptide GX1), and biotin-URP (biotinylated peptide URP) were synthesized by GL Biochem (Shanghai, China), and the peptides were analyzed by mass spectrometry and HPLC at GL Biochem. All peptides were preserved at −20 °C after the freeze-drying process.

### Co-immunoprecipitation

Co-immunoprecipitation (Co-IP) was performed as previously described^27^. Total cell lysates for each sample were collected from three 10-cm plates of co-HUVECs and was incubated with 100 μl of prewashed M-280 Streptavidin Dynabeads (Invitrogen, Catalog Number: 11205D, USA) at room temperature for 6 h. The tube was later placed into a magnetic stand to collect the beads against the side of the tube at this stage and during subsequent steps. The beads were conjugated with a nonspecific binding protein, diluted with 40 μl of RIPA lysis Buffer (Beyotime, Catalog Number: P0013B, China) containing protease inhibitor (Roche, Catalog Number: 04693159001, Switzerland) and preserved as a negative control. The supernatant was collected and incubated overnight at 4 °C with biotin-labeled URP that had been pre-conjugated to Dynabeads M-280 Streptavidin at room temperature for 30 min. The beads-URP-nonspecific binding protein compounds were collected and diluted with 40 μl of RIPA lysis Buffer (containing protease inhibitor,) and preserved as a negative control. The supernatant was then collected and incubated at 4 °C for 36 h with biotin-labeled GX1 that had been pre-conjugated to Dynabeads M-280 Streptavidin at room temperature for 30 min. The beads-GX1-specific binding protein compounds were collected and diluted with 40 μl of RIPA lysis Buffer (containing protease inhibitor) and were co-IP products. All collected protein complexes were eluted with 10 μl of 5 × loading buffer by boiling for 5 min and the eluates were subjected to SDS-PAGE.

### Coomassie blue G250 staining

After co-immunoprecipitation, equal amounts of proteins were loaded in 10% SDS-PAGE gels and electrophoresed at 25 mA for 50 min. Gels were stained with Coomassie blue G250 (BIO-RAD, Catalog Number: 1610406, USA) according to the manufacturer’s instructions.

### Protein extraction and Western blotting

As described previously^28,29^, proteins in cell lysates were extracted using RIPA lysis buffer with the protease inhibitor (Roche, Catalog Number: 04693159001, Switzerland). Protein concentrations were measured using the BCA protein assay kit (Beyotime, Catalog Number: P0010, China) according to the manufacturer’s instructions. Equivalent amounts of protein (30 μg) were separated on 10% SDS-PAGE gels and later transferred onto 0.2 μm nitrocellulose membranes (GE Amersham Protran, Catalog Number: 10600001, USA) according to the standard protocols. Membranes were blocked with 5% milk in TBST buffer for 1 h at room temperature, followed by incubation with primary antibodies at 4 °C overnight. After incubation with a secondary antibody for 1 h at room temperature, proteins were visualized using an ECL regent (Thermo Fisher Scientific, Catalog Number: 34094, USA). The primary antibodies were as follows: β-actin (1:2000, Sigma- Aldrich, Catalog Number: A1978, USA), botin-GX1(0.1 mg/ml, synthesized by GL Biochem), biotin-URP (0.1 mg/ml, synthesized by GL Biochem), TGM2 (0.5 μl/ml, RD, Catalog Number: AF4376, USA), Caveolin-1(1:500, ImmunoWay, Catalog Number: YT0686, USA), NF-κB (1:500, ImmunoWay, Catalog Number: YT3108, USA) and HIF1α (1:500, Millipore, Catalog Number: MAB5382, USA). The secondary antibodies were as follows: HRP-conjugated streptavidin, (1:1000, Bioss, Catalog Number: bs-0437P-HRP, China), peroxidase-conjugated AffiniPure goat anti-mouse IgG (1:5000, ZSGB-BIO, Catalog Number: ZB-5305, China), peroxidase-conjugated AffiniPure goat anti-rabbit IgG (1:5000, ZSGB-BIO, Catalog Number: ZB-2301, China) and sheep IgG horseradish peroxidase-conjugated antibody (1:1000, RD, Catalog Number: HAF016, USA).

### Mass spectrum analysis

According to the protocol described by Kang, J^27^, the protein bands of interest were excised from Coomassie blue-stained gel. Each gel slice was diced into small pieces (1 mm * 1 mm) and placed into a 1.5-ml tube. A gel piece that was removed from a protein-free region of the gel was used as a parallel control. Sample preparation used for Q-Exactive mass spectrometry was performed according to the standard protocol as described previously^30^. Gel slices were destained and digested in 20 μl of sequencing grade trypsin at 37 °C overnight. The protein digests were later desalted for MS and MS/MS analysis, which were performed in our lab using the Q-Exactive system (Thermo Fisher Scientific, USA). Afterward, Proteome Discoverer software (version 1.4; Thermo Fisher Scientific, USA) was applied for protein identification and quantitation.

### Cell Counting Kit-8(CCK-8) test

Co-HUVECs proliferation with or without TGM2 downregulation was determined by CCK-8 (DOJINDO, Catalog Number: CK04, Japan) assay as follows: cells in log-phase were seeded in 96-well plates at a density of 5000/well, and after 48 h, 10 μl of CCK-8 reagent was added to each well, with subsequent continued incubation at 37 °C for 2.5 h. Then optical density values were measured and analyzed.

### Flow cytometry (FCM) assay

The apoptosis of co-HUVECs was detected by FCM analysis as described^29,31^. The Annexin V-FITC apoptosis detection kit (BD Biosciences, Catalog Number: 556547, China) was used for apoptosis assays. Cells (1 × 10^4^) were incubated with GX1(0.1 mg/ml) for 48 h, stained according to the manufacturer’ s protocol, and sorted using a fluorescence-activated cell sorter (BD, China), and the data were analyzed using MODFIT software (BD, China).

### Immunofluorescence (IF) staining

According to previously described method^32^, HUVECs and co-HUVECs were seeded into Millicell EZ SLIDE (Millipore, Catalog Number: R6MA99969, USA). After cell attachment, cells were first fixed with 4% paraformaldehyde (LEAGENE, Catalog Number: DF0135, China) for 15 min at room temperature. After rinsed with phosphate buffered saline (PBS, HyClone, Catalog Number: AC12557265, USA), cells were blocked in block solution (ZSGB-BIO, China) for 30 min at 37 °C. Then, cells were incubated with the appropriate primary antibody overnight at 4 °C. After being rinsed with PBS, the cells were incubated with corresponding fluorescent secondary antibodies for 30 min at room temperature. After being rinsed with PBS, cells were incubated with DAPI for 15 min at room temperature and subsequently examined by confocal microscopy (Olympus, Japan). The primary antibody concentrations were as follows: botin-GX1 (0.1 mg/ml, synthesized by GL Biochem), biotin-URP (0.1 mg/ml, synthesized by GL Biochem), and anti-TGM2 (1:100, Abcam, Catalog Number: ab2386, UK). The fluorescent secondary antibody dilutions were as follows: fluorescein-conjugated goat anti-mouse IgG (1:50, ZSGB-BIO, Catalog Number: ZB-0312, China) and Cy5-streptavidin (1:160, BioLegend, Catalog Number: 405205, USA). DAPI (1:100, Bioworld, Catalog Number: BS5010, USA) and Vybrant DiD cell-labeling solutions (1:200, Invitrogen, Catalog Number: V22889C, USA) were used adhering strictly to the manufacturer’s instructions.

As described previously^33^, to examine the co-localization of GX1-receptor and TGM2 (Fig. 3c), cells were fixed, blocked and incubated with primary antibody solution (a mix of biotin-GX1 and anti-TGM2 in PBS) over night at 4 °C. After being rinsed with PBS, cells were incubated with fluorescent secondary antibody solution (a mix of fluorescein-conjugated goat anti-Mouse IgG and Cy5-streptavidin in PBS). After being rinsed with PBS, cells were incubated with DAPI for 15 min at room temperature and examined by confocal microscopy.

To examine the effect of GX1 on the subcellular distribution of TGM2 (Fig. 7b), live cells pre-incubated with DiD for 1 h at 37 °C before fixation with paraformaldehyde. Then, cells were fixed with 4% paraformaldehyde for 15 min at room temperature. After being rinsed with PBS, cells were blocked in block solution for 30 min at 37 °C. Then, cells were incubated with anti-TGM2 overnight at 4 °C. After being rinsed with PBS, cells were incubated in fluorescein-conjugated goat anti-mouse IgG for 30 min at room temperature. After being rinsed with PBS, cells were incubated with DAPI for 15 min at room temperature and examined by confocal microscopy.

### Immunohistochemistry (IHC) staining

Serial GC tissue sections were obtained from Xi’jing hospital, and the GC tissue microarray was purchased from SOBC (Catalog Number: HStmA180Su09, China). Notably, the serial GC tissue sections were cut continuously from the same paraffin blocks of GC patients to examine the co-localization of GX1-receptor, CD31(vascular marker) and TGM2. The IHC procedure was performed as described by Wang G^28^. Three serial sections were incubated with biotin-GX1, anti-CD31 or anti-TGM2. The primary antibody concentrations were as follows: botin-GX1 (0.1 mg/ml, synthesized by GL Biochem), anti-CD31 (1:50, Abcam, Catalog Number: ab28364, USA) and anti-TGM2 (1:50, Abcam, Catalog Number: ab2386, USA). The secondary antibodies were as follows: HRP-conjugated streptavidin (1:1000, Bioss, Catalog Number: bs-0437P-HRP, China), peroxidase-conjugated AffiniPure goat anti-mouse IgG (1:5000, ZSGB-BIO, Catalog Number: ZB-5305, China), peroxidase-conjugated AffiniPure goat anti-rabbit IgG (1:5000, ZSGB-BIO, Catalog Number: ZB-2301, China). The immunostaining intensity was scored on a scale of 0 to 3: 0 (negative), 1 (low), 2 (moderate) and 3 (high). The percentage of positive cells was evaluated on a scale of 0 to 4: 0 (0%), 1 (1–25%), 2 (26–50%), 3 (51–75%), and 4 (76–100%). The final immuno-activity scores were calculated by multiplying the above two scores, resulting in an overall score that ranged from 0–12. Each case was ultimately considered “Low” if the final score ranged from 0–3 and “High” if the final score ranges from 4–12.

### Transfection of co-HUVECs with siRNAs

To transfect co-HUVECs with siRNA, Lipofectamine 2000 (Invitrogen, Catalog Number: 11668019, USA) was used according to the manufacturer’s instructions. Next, co-HUVECs were transfected with three siRNAs (Gene Pharma) to decrease the expression of TGM2. After detection of TGM2 expression at mRNA and protein levels (Supplementary Fig. 1), we finally chose siRNA-TGM2-1 (F: 5′ GCUACCAGGGAUCCAGC-UUTT3′, R: 5′AAGCUGGAUCCCUGGUAGCTT3′) and siRNA-TGM2-2 (F: 5′ CCAAGUACGAUGCGCCCUUTT3′, R: 5′AAGGGCGCAUCGUACUUGGTT3′) to downregulate the expression of TGM2 in co-HUVECs and we chose siRNA-NC (F: 5′UUCUUCGAACGUGUCACGUTT3′, R: 5′ ACGUGACACUUCGGAGAATT3′) as a negative control.

### RNA extraction and real-time PCR (RT-PCR) assay

As described previously^34,35^, total RNA from cell lines was extracted using aa TaKaRa MiniBEST universal RNA extraction kit (TaKaRa, Catalog Number: 9767, Japan) per the manufacturer’s instructions. PCR primers for GAPDH, NF-κB and HIF1α were purchased from TaKaRa and those for TGM2 from Sangon Biotech(China). The PCR primers for GAPDH were 5′GCACCGTCAAGGCTGAGAAC3′ (Forward) and 5′TGGTGAAGACGCCAGTGGA3′ (Reverse). The primers for NF-κB were 5′GCCTCCACAAGGCAGCAAATA3′ (Forward) and 5′CACCACTGGTCAGAGACTCGGTAA3′ (Reverse). The primers for HIF1α were 5′CTCATCAGTTGCCACTTCCACATA 3′ (Forward) and 5′AGCAATTCATCTGTGCTTTCATGTC 3′ (Reverse). The primers for TGM2 were 5′ACCGCTGAGGAGTACGTCTG3′ (Forward) and 5′CAGAGAAAGGCTCCAGGTTG3′ (Reverse). cDNA was synthesized using a PrimeScript RT reagent kit (TaKaRa, Catalog Number: RR036A, Japan) and real-time PCR was performed using the SYBR premix Ex Taq II (TaKaRa, Catalog Number: RR820A, Japan). Fluorescence was measured in a LightCycler 480 system (Roche, Switzerland). GAPDH was used as the internal control for mRNA measurements. Each sample was run in triplicate.

### In vitro cell migration assay

Transwell and wound healing assays were conducted to measure migratory ability of co-HUVECs. For Transwell assays, a 24-well Transwell plate (8-μm pore size, Corning, USA) was used as described previously^36^. A total of 2 × 10^4^ co-HUVECs were seeded to the top chamber of the Transwell. Cells were suspended in medium with GX1 (0.1 mg/ml) without serum or growth factors, and medium supplemented with serum was used as a chemoattractant in the lower chamber. After incubation at 37 °C for 24 h, the top chambers were wiped with cotton wool to remove the non-migratory cells. The invading cells on the underside of the membrane were fixed in 100% methanol for 10 min, air-dried, stained with 0.1% crystal violet, and counted under a microscope. For wound healing assays, the Culture-Insert 2 Well (ibdi, Catalog Number: 80206, Germany) was used. First, 70 μl of co-HUVEC suspension (5 × 10^5^ cells/ml) was added into each well, and then, after cell attachment (24 h later), the Culture-Insert 2 Well was gentle removed with sterile tweezers. Next, the used dished was filled with TCM free of FBS. Pictures were captured every 12 h and subsequently analyzed with ImageJ software.

### Pre-embedding immunogold-silver cytochemistry

Immunoelectron microscopy was conducted to monitor the effect of GX1 on the subcellular distribution of TGM2 in co-HUVECs as described previously^37,38^. Co-HUVECs were treated with 0 or 0.1 mg/ml GX1 for 24 h. After trypsinization and centrifugation for 15 min at 1000 rpm and 4 °C, the supernatants were discarded. The pellets were fixed with 4% paraformaldehyde and 0.05% glutaraldehyde in phosphate buffered saline (PBS) for 2 h at 4 °C and then rinsed with PBS for 30 min. Next, the pellets were blocked with blocking solution (5% bovine serum albumin (BSA) and 0.05% Triton X-100 in PBS) for 3 h at room temperature and then rinsed with PBS for 15 min. The pellets were incubated for 24 h at room temperature with a rabbit anti-TGM2 primary antibody (1:100, Abcam, Catalog Number: ab109200, UK) diluted in PBS containing 1% BSA and 0.05% Triton X-100, and the pellets then rinsed with PBS for 30 min. Next, the pellets were incubated with secondary antibody comprising anti-rabbit IgG conjugated to 1.4 nm gold particles at a 1:100 dilution (Nanoprobes, USA). After rinsing with PBS for 30 min, pellets were postfixed with 2% glutaraldehyde in PBS for 1 h. Silver enhancement was performed in the dark with the HQ Silver Kit (Nanoprobes) for the visualization of TGM2 immunoreactivity. Before and after the silver enhancement step, pellets were rinsed with de-ionized water for 10 min. After rinsed with 0.1 M phosphate buffer solution (PB), immuno-labeled pellets were fixed with 0.5% osmium tetroxide in 0.1 M PB for 1 h at room temperature, dehydrated in a graded ethanol series and propylene oxide, and flat-embedded in Epon 812 between sheets of plastic. After polymerization, the flat-embedded pellets were trimmed and glued onto blank resin stubs. Serial ultrathin sections were cut with an Ultramicrotome (Leica EM UC6) using a diamond knife (Diatome, PA) and mounted on formvar-coated mesh grids (6–8 sections/grid). They were then counterstained with uranyl acetate and lead citrate and observed under a JEM-1230 electron microscope (JEOL LTD, Japan) equipped with a CCD camera and its application software (Gatan, Warrendale, PA). Electron micrographs were arranged and contrast-enhanced with a computer.

### TGM2 GTP binding assay

The GTP-binding/GTP-agarose pull-down assay was performed to detect the effect of GX1 on the GTP-binding activity of TGM2 as described^23^. Co-HUVECs were treated with 0 or 0.1 mg/ml GX1 for 24 h. After trypsinization, the cells were rinsed in ice-cold PBS, pelleted and resuspended in GTP-binding buffer containing 20 mM Tris-HCl, pH = 7.5, 5 mM MgCl_2_, 2 mM PMSF, 20 μg/ml leupeptin, 20 μg/ml pepstatin, 10 μg/ml aprotinin plus 150 mM NaCl and 0.1% Triton X. The cells were sonicated for 15 s and centrifuged at 13,000×*g* for 10 min at 4 °C, and the supernatant was collected. A fraction of the supernatant was set aside for electrophoresis to determine the total TGM2 level. Supernatant protein (100 μg) was incubated with 100 μl of GTP-agarose beads (Sigma-Aldrich, Catalog Number: G9768, USA), in a total volume of 500 μl of GTP-binding buffer for 30 min at 4 °C. The beads were centrifuged at 10,000×*g* for 2 min, and the supernatant was retained. The beads were washed three times with 1 ml of GTP-binding buffer, and the retained supernatant was incubated with the beads for another 30 min. The beads were washed and incubated with the retained supernatant overnight at 4 °C. The beads were then washed seven times with GTP-binding buffer, and bound protein was eluted by boiling in 100 μl of 2 × Laemmli buffer. The samples (50 μg protein equivalents, 50 μl) were then electrophoresed for anti-TGM2 immunoblot. The total supernatant (50 μg of protein) was electrophoresed in parallel.

### Statistical analysis

All experiments above were repeated at least three times. Statistical analysis was performed by GraphPad Prism 7.03 software. All data were expressed as the mean ± S.D. Student’s *t*-test was used to analyze the differences between the means. *P* < 0.05 was statistically significant.

## Electronic supplementary material

Supplementary table 1

Supplementary table 2

Supplementary figure legends

Supplementary figure 1

Supplementary figure 2
