# Supplementary material for: Novel peptide GX1 inhibits angiogenesis by specifically binding to transglutaminase-2 in the tumorous endothelial cells of gastric cancer
Source: Cell Death Dis. 2018 May 21;9(6):579. doi: 10.1038/s41419-018-0594-x (PMC5962530; doi:10.1038/s41419-018-0594-x)
Supplement: Supplementary file 1 — Supplementary table 1 [file 41419_2018_594_MOESM1_ESM.docx]

Supplementary Table 1 List of candidate receptors of GX1 that were identified by co-immunoprecipitation and mass spectrometry (twice co-immunoprecipitation results exhibited below). GX1 immunoprecipitation samples were detected to identify the candidate receptors of GX1 by mass spectrometry and URP immunoprecipitation samples acted as a negative control. IP-GX1 and IP-URP represented proteins binding to GX1 and URP respectively. Protein IDs, gene name, protein name, molecular weight and peak areas of the total 55 proteins were listed in the table, and the proteins were ranked according to the size of molecular weight. In terms of the GX1-receptor molecular weight (~70 kDa), specifically binding to GX1 but not URP and repeatability, TGM2 was eventually screened from 55 proteins as the candidate receptor of GX1 targeting to gastric cancer.
