# Supplementary material for: Novel peptide GX1 inhibits angiogenesis by specifically binding to transglutaminase-2 in the tumorous endothelial cells of gastric cancer
Source: Cell Death Dis. 2018 May 21;9(6):579. doi: 10.1038/s41419-018-0594-x (PMC5962530; doi:10.1038/s41419-018-0594-x)
Supplement: Supplementary file 2 — Supplementary table 2 [file 41419_2018_594_MOESM2_ESM.docx]

**Supplementary Table 2:** Comparison between the receptor of GX1 and TGM2

| **Name** | **Receptor of GX1** | **TGM2** |
| --- | --- | --- |
| **Molecular weight** | Approximately 70 kDa | 76 kDa |
| **Subcellular localization** | Cytosol, plasma membrane | Cytosol, plasma membrane |
| **Tissue distribution** | High expression in gastric cancer blood vessels | Elevated expression in gastric cancer |
| **Function** | 1.Inhibiting angiogenesis  2.Inducing apoptosis of endothelial cell | 1.Regulation of cell growth and apoptosis  2.Promoting angiogenesis  3.Mediation of cell adhesion  4.Modification of ECM |
