# Supplementary material for: Novel peptide GX1 inhibits angiogenesis by specifically binding to transglutaminase-2 in the tumorous endothelial cells of gastric cancer
Source: Cell Death Dis. 2018 May 21;9(6):579. doi: 10.1038/s41419-018-0594-x (PMC5962530; doi:10.1038/s41419-018-0594-x)
Supplement: Supplementary file 3 — Supplementary figure legends [file 41419_2018_594_MOESM3_ESM.docx]

Supplementary Figure 1 screening of siRNAs to downregulate TGM2 expression. We had achieved three siRNAs with different sequences (siRNA-TGM2-1: F: 5’GCUACCAGGGAUCCAGCUUTT3’, R: 5’AAG CUGGAUCCCUGGUAGCTT3’; siRNA-TGM2-2: F: 5’ CCAAGUACGAUGCGCCCUUTT3’, R: 5’ AAGGGCGCA UCGUACUUGGTT 3’; siRNA-TGM2-3: F: 5’GCAGUGACUUUGACGUUTT3’, R: 5’AAGACGUCAAAGUCACUGCTT3’) and a negative control sequence (siRNA-NC: F: 5’UUCUUCGAACGUGUCACGUTT3’, R: 5’ACGUGACACUUCG GAGAATT3’) from Gene Pharma company. Then qRT-PCR (A) and western blotting assays (B) were performed to measure the interference efficiency. According to the results of qRT-PCR and western blotting, we found that the interference efficiency of siRNA-TGM2-1 and siRNA-TGM2-2 was better than siRNA-TGM2-3, so we eventually chose siRNA-TGM2-1 and siRNA-TGM2-2 to interfere TGM2 expression in co-HUVECs.

Supplementary Figure 2 GX1 inhibits the expression of NF-κB and HIF1α with dose-dependent effects. Western blotting was conducted to detect the expression of NF-κB and HIF1α in co-HUVECs pre-incubated with different concentrations of GX1 (0, 0.05, 0.075, 0.1, 0.125 and 0.15 mg/ml; 24 hours). Analysis of the result showed that the expression of NF-κB and HIF1α were suppressed significantly with dose-response effects simultaneously.
