# Supplementary figures and images for: Novel peptide GX1 inhibits angiogenesis by specifically binding to transglutaminase-2 in the tumorous endothelial cells of gastric cancer
Source: Cell Death Dis. 2018 May 21;9(6):579. doi: 10.1038/s41419-018-0594-x (PMC5962530; doi:10.1038/s41419-018-0594-x)

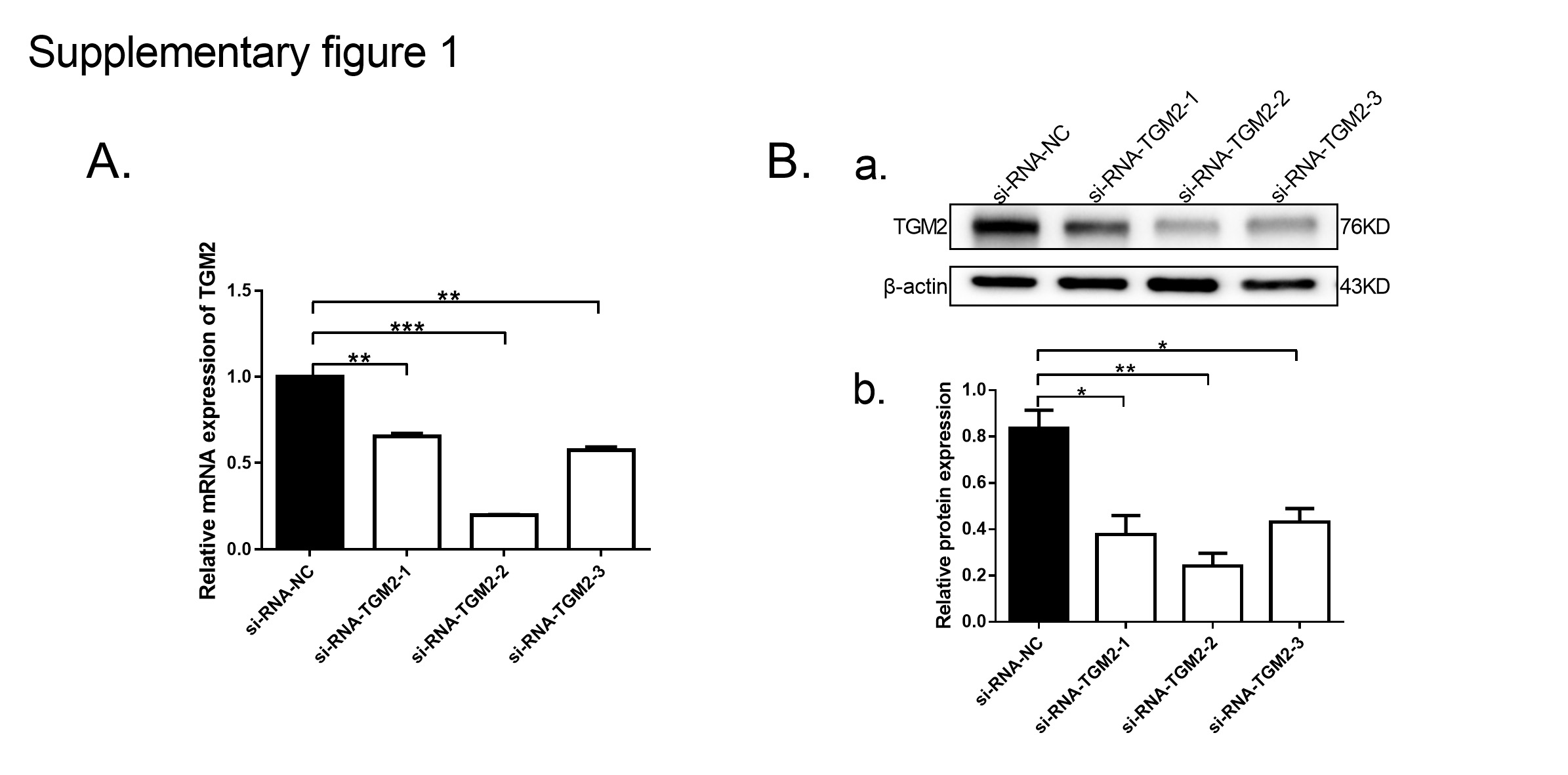

Supplement: Supplementary file 4 — Supplementary figure 1 [file 41419_2018_594_MOESM4_ESM.jpg]

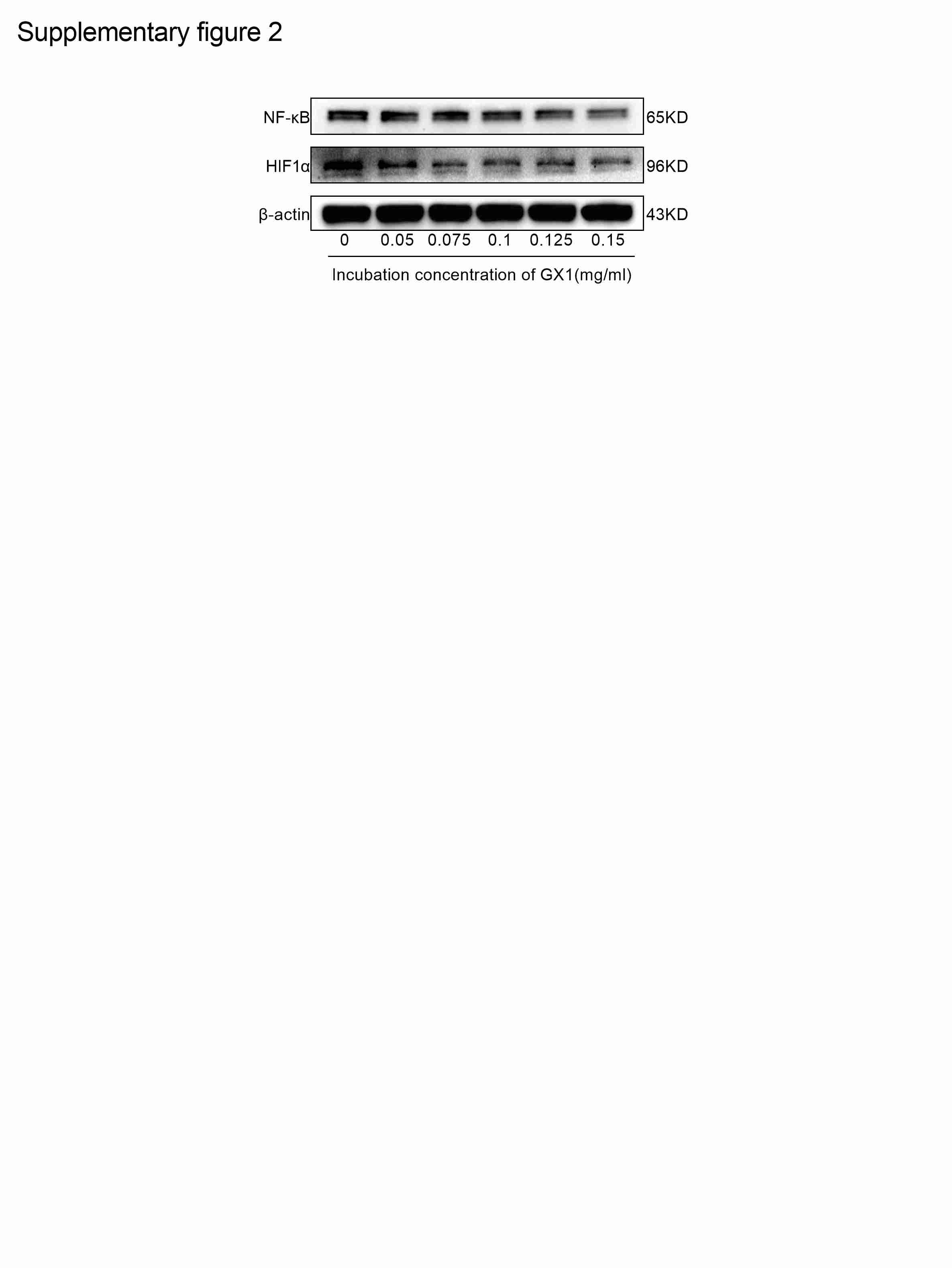

Supplement: Supplementary file 5 — Supplementary figure 2 [file 41419_2018_594_MOESM5_ESM.jpg]
